# Moving Health Literacy Research and Practice towards a Vision of Equity, Precision and Transparency

**DOI:** 10.3390/ijerph17207650

**Published:** 2020-10-20

**Authors:** Shuaijun Guo, Xiaoming Yu, Orkan Okan

**Affiliations:** 1 Centre for Community Child Health, Murdoch Children’s Research Institute, Royal Children’s Hospital, Melbourne, VIC 3052, Australia; 2 Department of Pediatrics, University of Melbourne, Melbourne, VIC 3010, Australia; 3 Institute of Child and Adolescent Health, School of Public Health, Peking University, Beijing 100191, China; yxm@bjmu.edu.cn; 4 Centre for Prevention and Intervention in Childhood and Adolescence (CPI), Faculty of Educational Science, Bielefeld University, 33615 Bielefeld, Germany; orkan.okan@uni-bielefeld.de

**Keywords:** health literacy, life course, precision public health, open science, knowledge translation

## Abstract

Over the past two decades, health literacy research has gained increasing attention in global health initiatives to reduce health disparities. While it is well-documented that health literacy is associated with health outcomes, most findings are generated from cross-sectional data. Along with the increasing importance of health literacy in policy, there is a lack of specificity and transparency about how to improve health literacy in practice. In this study, we are calling for a shift of current research paradigms from judging health literacy levels towards observing how health literacy skills are developed over the life course and practised in the real world. This includes using a life-course approach, integrating the rationale of precision public health, applying open science practice, and promoting actionable knowledge translation strategies. We show how a greater appreciation for these paradigms promises to advance health literacy research and practice towards an equitable, precise, transparent, and actionable vision.

## 1. Introduction

Health literacy underpins everyday health behaviours and health-related decisions. Defined as an individual’s ability to find, understand, and use health information to promote and maintain good health [1,2], the term “health literacy” has been widely used in healthcare, disease prevention, and health promotion since the 1990s [3]. Health literacy is a context- and content-specific concept; this means that researchers need to define and measure it within a specific context for a particular purpose [4]. From a public health perspective, health literacy is regarded as a personal asset that evolves over the life course and promotes empowerment in health decision-making [5,6,7]. In the context of the coronavirus disease of 2019 (COVID-19), an individual’s health literacy supports his/her decisions on washing hands, maintaining physical distance, adopting protective behaviours, seeing a doctor, and complying with quarantine policies, thus contributing to a more likely successful public health response strategy [8,9,10]. Health literacy also helps to navigate the infodemic—the overabundance of valid and invalid information that is circulating on the Internet—that is attached to the COVID-19 pandemic [11,12].

Low health literacy is a global public health concern. Internationally, it is estimated that 28.7% to 92.7% of adults have low health literacy [13,14], costing national governments at least $106 billion annually [15]. Mounting evidence suggests that low health literacy is associated with adverse health outcomes [16,17,18], including frequent use of emergency care, prolonged hospital stays, and high mortality rates, which in turn lead to health disparities [19]. National and international health programs have shown promising outcomes (e.g., improved health knowledge, healthier behaviours, self-management of chronic illness, access to healthcare) when intervening to improve health literacy [20,21,22]. Most recently, the World Health Organization’s Shanghai Declaration on Promoting Health in the 2030 Agenda for Sustainable Development highlighted health literacy as an integral part of the skills developed over a lifetime and recognized it as a critical driver of achieving an equitable world [23,24].

## 2. What Is Known Already?

Enhancing health literacy requires a systems approach to understanding its risk factors and its impact on health outcomes [4,25,26]. The social determinants framework suggests that health literacy is an interactive product of an individual’s health literacy skills and the broad environment and culture [27,28]. Empirical studies show that health literacy levels differ substantially across age groups and countries. Based on the Demographic and Health Surveys, McClintock et al. [29] found that the prevalence of poor health literacy among respondents aged 15–49 years ranged from 36.1% in Namibia to 91.5% in Niger across 14 sub-Saharan countries. As for children and adolescents, the Health Behaviour in School-aged Children study shows that, in 10 countries (e.g., Austria, England, Finland), a total of 13.3% of participants have low levels of health literacy, ranging from 6.0% to 17.7% across countries [30].

There is a social gradient in health literacy for children [31], adolescents [32], and adults [14]. The lower the socioeconomic status an individual has, the lower the health literacy level is likely to be. Health literacy can affect health outcomes at each life stage. Prior to childbirth, low health literacy in pregnant mothers has a significant impact on the health and development of their offspring, including prematurity, infancy death, and child vaccination participation [33]. Low health literacy in children and adolescents is associated with poor health behaviours, such as smoking, alcohol use, and obesity [34,35,36,37]. When children and adolescents transit into adulthood and older age, health literacy is closely linked with healthcare outcomes, such as prolonged hospitalization and poor medication adherence [17,38].

While health literacy research has gained momentum in the global context [39,40], it is predominated by cross-sectional studies, with less than 8% of all published papers focusing on health literacy interventions, including randomized controlled trials [41,42]. Unlike time-series data, cross-sectional data make it impossible to make a valid statement regarding the change. Health literacy is a life-course personal asset [43], which progresses as a child grows up with different characteristics and health needs at each life stage [7,44]. For instance, children’s and adolescents’ health literacy rely heavily on their developmental ability and their parents and peers [45]. When they transition into adulthood, they become more independent in making their own decisions in healthcare, disease prevention, and health promotion [14]. As cognitive function declines with age, older adults are an especially vulnerable group, with low self-management ability for everyday health-related decisions [43,46,47]. Currently, there is a lack of holistic ways to look at the impact of health literacy over the life-course due to a lack of longitudinal studies.

There is promising evidence showing the effectiveness of health literacy interventions on health outcomes at the individual and community level [20,48,49,50]. However, there remain substantial gaps. In practice, health literacy interventions vary in terms of their study designs, measurement tools, and types of outcome measures [21,22]. Besides, there is a lack of specificity in the intervention targets (e.g., individual level, organizational level, community level), content (e.g., functional health literacy, interactive health literacy, critical health literacy), timing (e.g., antenatal, preschool, adolescence), and formats (e.g., universal, intensive, low-threshold). It remains unclear about which interventions are the most effective in improving health literacy, related health behaviours, and associated health outcomes. When translating health literacy evidence into practice, researchers should design interventions that are specifically tailored to people with different health literacy levels and needs [21,51]. There is a need to use precise and transparent approaches to improving health literacy and reducing health inequities in the end.

In response to low health literacy levels in the population, many countries have developed national action plans to strengthen health literacy for achieving sustainable development and health equity (e.g., the National Actional Plan to Improve Health Literacy in the USA [52], the National Statement on Health Literacy in Australia [53], the National Action Plan Health Literacy in Germany [54]). Common features in these policy documents include a response to perceived deficiencies in health literacy, the importance of professional education in improving the quality of communication, and a need for responsive healthcare systems [55,56]. Policy responses to health literacy are important public statements of priorities by governments and provide a mechanism for public accountability [55,57]. However, in contrast to the increasing number of evidence generated from empirical studies, discussions on the knowledge translation and implementation process are scarce. There remains a lack of specificity in the implementation process and monitoring systems for progress.

## 3. What Evidence Is Needed?

This perspective is a proposition for four new research paradigms to address the aforementioned knowledge gaps, expecting to move health literacy research and practice towards an equitable, precise, transparent, and actionable vision. This includes using a life-course approach to health literacy [58], integrating the rationale of precision public health [59], applying open science practice [60], and promoting actionable knowledge translation strategies [61]. In what follows, we will discuss the life-course approach to health literacy as a starting point, and then the necessity of integrating the rationale of precision public health. We are calling for a shift of current research paradigms from judging health literacy levels (low versus high) towards observing how health literacy skills are practised and developed over the life-course. Based on these new paradigms, we expect a nuanced understanding of how health literacy develops over the life-course, how it influences health behaviour and decision-making, and thus how it informs specific interventional opportunities—especially in the early life stages across educational and healthcare settings—for a precise policy recommendation. We also highlight the importance of applying open science and considering knowledge translation strategies from the beginning of research planning to generate or replicate policy-relevant findings rapidly and cost-effectively across different cultural contexts, and thus facilitate the process of knowledge dissemination.

### 3.1. A Life-Course Approach to Health Literacy

**Figure 1:**
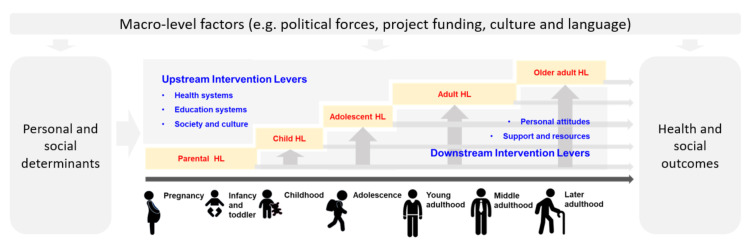
A life-course approach to health literacy (HL) and its impact on health and social outcomes.

We need to extend the current concept of “health literacy” from cross-sectional to longitudinal studies. Health literacy is a personal asset that develops dynamically over time [43]. A life-course approach to health literacy will assist researchers in discovering opportunities for optimizing health development and reducing health inequities, and explaining how health practices and policies can go beyond the avoidance of disease to the promotion of health at the early life stages [7,42,43]. As shown in Figure 1, we recognize potential intervention levers (both upstream and downstream) for giving all children the best start to life. A life-course approach to health literacy aligns with national and international health initiatives that aim to reduce inequities (e.g., the National Action Plan for Children and Young People in Australia [62]).

A life-course approach is well-recognized in public health research and practice to close the gap in health inequities [58,62]. Using life-course data from the Wisconsin Longitudinal study 1957–2011, Clouston et al. [43] found that life-course predictors of health literacy among older adults included parental educational attainment, and adolescent cognitive and non-cognitive skills. Findings from this life-course analysis add to our understanding of how health literacy might change over time through adolescent cognitive and non-cognitive skills. Depending on the research purpose and available data sources, researchers could also propose other specific research questions using one of the life-course models exemplified in Table 1. For example, early life represents a sensitive period of health and development. Exposure to stressors associated with disadvantages during this time can exert adverse effects on health over the life course [7]. Using the sensitive period model, researchers can examine and compare the effect of parental health literacy on children’s health behaviours and health outcomes at different ages of children (e.g., pregnancy, infancy, toddler age).

### 3.2. Precision Public Health and Health Literacy

We are entering an era of “big data” and “precision”. Big data has enabled extensive and specific research and trials of stratifying and segmenting populations at risk for a variety of health problems, including poor health literacy [67]. In the field of big data and public health, machine learning is a fundamental component of data analytics that provides data-driven insights, decisions, and predictions [68,69]. Machine learning techniques have been broadly adopted for researchers to answer a series of public health research questions (e.g., identifying leading dietary determinants in children [70], predicting the development of Type 2 Diabetes [71]). Using different machine learning approaches, researchers can also address health literacy research questions, such as identifying elderly people at high risk of low health literacy.

Particularly, the breadth of longitudinal data available in existing cohorts enables researchers to generate policy-relevant findings quickly [72]. Similar to the precision medicine initiative of providing the right treatment to the right patient at the right time [73], a precision public health approach to health literacy calls for harnessing the power of resourceful life-course data to inform the right intervention to the right population at the right time [59,74]. In the context of COVID-19 [75], precision public health is particularly useful to design targeted interventions for populations by person, place, and time to promote better navigation of health care and disease prevention [76]. If a population has a higher proportion of persons with low health literacy, public messages could be provided to educate persons on where to obtain trustworthy information and when to seek health professionals [76].

Integrating the rationale of precision public health aligns with the relation- and context-specific nature of health literacy [4,26]. Currently, there is a lack of specificity to inform clear health literacy policy decisions [72]. Figure 1 shows that there are substantial opportunities for researchers to generate specific recommendations between personal and social determinants and health literacy (i.e., upstream intervention levers), and between health literacy and health and social outcomes (i.e., downstream intervention levers). For example, the education sector is a critical platform for health literacy interventions, and education for health literacy is a fundamental process and outcome across the life course [40]. Precision evidence is needed, such as at which time point, at what dosage, and which delivery approach is likely to have the most significant impact on improving population health literacy and reducing health inequities. We need to identify precise policy levers (either upstream or downstream) and build an evidence base with sufficient specificity to generate actionable policy implications.

### 3.3. Open Science Practice and Health Literacy

Open science refers to a range of practices that promote transparency, openness, and reproducibility in research [77]. Efforts to reproduce published findings have yielded a concerningly high failure rate (e.g., only 62% replicated in Nature and Science [78]) [79,80]. In response to concerns about this “reproducibility crisis”, the open science practice has been increasingly recognized across disciplines [60]. For instance, the National Health and Medical Research Council and the Australian Research Council have clear open access policies that align with the Australian Government’s commitment to open access and open data management [81]. However, in practice, null results are less frequently published than statistically significant results and are more likely to be inaccessible and lost in the “file drawer” [82]. To reduce publication bias, we need to move the current evidence of health literacy from an era of “publish or perish” to “visible or vanish” [83].

Transparency, openness, and reproducibility are central principles of open science practice [77]. Examples of open science practice include a preregistered report, detailed analytic plan, and publicly shared coding via the Open Science Framework (Table 2) [77,84]. A future vision for health literacy research is to increase its clarity, credibility, and transparency, which can help to provide reliable evidence that can serve as a basis for making decisions about clinical or population-health interventions [85]. For example, the Health Literacy Tool Shed is an online, publicly accessible database of health literacy measures [86]. Currently, more than 200 measurement tools are available. Healthcare providers and researchers can search and select the most appropriate instrument according to a specific research purpose [86]. Adoption of open science practice in health literacy research is effective to replicate studies across different cultural contexts. It also provides researchers with a system structure in documenting their work and improving workflows, and offers a path to publication irrespective of the null conclusions [84,87].

### 3.4. Knowledge Translation and Health Literacy

Knowledge translation is the exchange, synthesis, and ethically sound application of research findings within a complex set of interactions among researchers and knowledge users [88]. While a number of knowledge translation frameworks has been developed for researchers [89], there is a well-known gap between research and practice [90,91]. It is estimated that it takes 17 years for just 14% of medical research to be implemented [83,92]. This is the same case in the field of health literacy [93,94]. While the importance of health literacy is increasingly recognized in national and global health initiatives [23,62,95], there is still a long way to go when applying health literacy into current practice [50,96]. The evidence synthesis shows that, of the 46 existing and developing health literacy policies in European regions, the main barriers influencing the successful implementation of health literacy policy include cultural barriers, budget restrictions, and the difficulty obtaining high-quality evidence. Besides, there is also a lack of engagement in policy evaluation by the academic community [20].

Translating the best available research evidence into evidence-based practice and policy is a complex process which confronts multiple barriers at the individual, organizational, and political level [97]. There has been a range of efforts to reduce these barriers. For example, the OPtimizing HEalth LIteracy and Access (Ophelia) is a whole-of-system approach to developing and implementing health literacy research [98]. This approach is widely accepted in high-income and low- and middle-income countries, and uses health literacy profiling and community engagement to create and implement health reforms, thus improving health and equity [98]. The Ophelia approach has also been adapted for different populations and contexts, such as the HealthLit4Kids [99]. Another well-established whole-of-system approach is organizational health literacy, which is widespread in North America and Europe [100]. Organizational health literacy is based on the assumption that health literacy is a relational concept in which not only individual skills must be addressed, as well as system-level complexities. This concept has also been used in the HeLit-Schools project, interlinking the organizational health literacy as applied to the school setting with the WHO health-promoting school framework [101,102].

## 4. Benefits and Challenge

### 4.1. Benefits

There are four main benefits if the above research paradigms are applied in current health literacy research and practice. First, we can monitor and evaluate population health literacy levels over time by implementing routine data collection. This allows us to look at health literacy levels among different age groups as well as vulnerable groups, such as those from different ethnic minorities, backgrounds, and migrants, children and young people, chronically ill, and older people. We can also examine the protective and risk factors of health literacy and its impact on health outcomes from a longer-term perspective, thus informing policy opportunities at the best time.

Second, we can investigate a specific research question about health literacy from a precision public health perspective. We can use modern epidemiological methods, such as causal inference to explore the ideal time point to intervene in low health literacy of a specific population [103,104]. When a randomized trial is not available, we can use the emulated target trial to investigate the causal effect of improving health literacy on a specific health outcome [105]. A most valuable approach to better understanding real-life health literacy is to focus on ethnographic research exploring the social practices when health information and knowledge are the action focus [106,107,108].

Third, through the open science movement initiative, it is cost-effective and time-efficient to measure, collect, and analyze health literacy data via existing or linked datasets. For example, the COVID Health Literacy Consortium (COVID-HL) is a timely project in the context of COVID-19 [109,110]. COVID-HL aims to establish a global network on digital health literacy and increase global awareness on health literacy as a critical tool to help protect from communicable diseases. This international platform makes it possible for a health literacy comparison across countries and enables collaborations and data access for researchers. Further examples include the WHO Action Network on Measuring Population and Organizational Health Literacy (M-POHL), which aims at the routine measurement of different types of health literacy in the European adult population [111].

Fourth, the knowledge translation and engagement process moves the generated health literacy evidence towards the real world. Knowledge end-users, such as policymakers or parents of young children, can benefit from interaction with researchers through reflections on their own daily activities, enhanced health knowledge, and skills to protect health. Researchers can also benefit as they gain a nuanced understanding of the practice and policy environment, and develop health literacy research questions that have real-world applicability and benefits [112].

### 4.2. Challenges

There are also several challenges. First, health literacy measurement is a complex phenomenon across the life stage, even at a particular time point. The assessment of health literacy varies depending on the setting, research purpose, and the scope of health literacy definitions [113,114]. Given that different age groups have different characteristics and health needs, researchers may consider using a core measurement tool plus a variety of add-on modules that target varying age groups [115]. Eventually, this will also make the measurement much more complex and time-consuming.

Second, it is complicated for life-course data planning and analysis using modern epidemiological methods. Dropouts, missing data, and other study deviations (e.g., low response rates) are a common occurrence in both population research and clinical studies [116]. It is important to consider the power analysis strategies to estimate the sample size, thus enabling researchers to detect a significant effect of health literacy on the outcome of interest. Researchers also need to consider critical questions commonly encountered in longitudinal data analysis, such as confounding bias, selection bias, measurement bias, and whether to include an interaction term in a parametric model [103]. In this case, informed by expert knowledge, researchers can use the directed acyclic graph [117] to visually represent the hypothesized causal pathway from health literacy to a specific health outcome. The LifeCourse analysis plan template is another useful toolkit that can strengthen the quality of observational epidemiological studies [118]. As for big data in public health, while it provides opportunities to make causality inferences based on chains of sequence, it also introduces challenges to machine learning, such as high data dimensionality, model scalability, and distributed computing [119].

Third, using open science practice in health literacy often requires more time and effort for archiving, documenting, quality controlling of codes, and data security [87]. Open science is changing how research and practice are conducted, and it takes time to consolidate in the mainstream [77]. Currently, the majority of open-source datasets do not adhere to data principles, such as being findable and accessible [120]. Besides, data access and sharing are recurring challenges attributed not only to privacy concerns, but also ambiguous data ownership and unaligned incentives [121]. There is a need for researchers to adhere to principles of research partnership and data governance models to prevent the breaches of privacy that obstruct ethically justified data access.

Fourth, knowledge translation barriers are common in practice, especially in the context of COVID-19 [93]. For example, when disseminating health literacy information in a multi-cultural setting, how could we engage with culturally and linguistically diverse families for the first time and get them to understand the right information? Along with challenges related to information overload and an ongoing infodemic [122,123], researchers and policymakers should be aware of the main facilitators that drive successful health literacy policy implementation, such as intersectoral working, political leadership, and overcoming cultural barriers [20]. In addition, specific knowledge translation plans are needed in advance when implementing relevant strategies in the real world [54,124,125]. There remains much work to be conducted to understand how to implement health literacy evidence into practice.

When applying the above paradigms into practice, researchers need to be aware that we are not calling for a “one size fits all” solution to fill the gaps in current research. Instead, we are calling for a more equitable, precise, transparent, and actionable way to advance health literacy in research and practice. The four paradigms mentioned above cover a broad range of considerations, ranging from a theoretical approach for individual research to empirical studies generating information using big data for policymaking. Researchers can integrate one or two into their research planning and implementation. For example, when a researcher is exploring personal experiences of health literacy at the micro levels, it is more suitable to consider using open science practice and knowledge translation strategies to enhance the rigour of reporting studies and disseminate research findings to a range of stakeholders.

## 5. Conclusions

Health literacy is a crucial driver to health equity. While the evidence base shows a significant impact of health literacy on health outcomes, we need to move this field towards an equitable, precise, transparent, and actionable vision. A life-course approach to health literacy will allow for a better understanding of the mechanisms linking health literacy to health outcomes. A precision public health rationale corresponds with the specific nature of health literacy, and will enable us to generate specific policy recommendations to improve population health. Open science practice will assist with minimizing publication bias and motivating researchers to share resources to produce more reliable and cost-effective evidence. Finally, actionable knowledge translation strategies will bridge the gap between the academic world and the real world, leading to an equitable society that is not so far away.

## Figures and Tables

**Table 1:** Applying life-course models to health literacy research.

Type of Models	Purpose	Example of Health Literacy Research Questions
The sensitive period model	To examine timing effects in which exposures during sensitive periods of development have stronger effects on health, social, emotional, and cognitive development outcomes than they would have at other life stages [63].	To examine and compare the effect of parental health literacy during pregnancy and infancy on infant and child health outcomes. To examine and compare the different timing effects of risk or protective factors (e.g., socioeconomic status) in early years on health literacy in later years.
The accumulation model	To examine the role of persistent advantage or disadvantage over time—in both specific life stages and over life stages—on health and development [64].	To examine the role of persistent advantage or disadvantage (e.g., socioeconomic status, ethnic minorities) on health literacy in a specific life stage and over the life course. To examine the effect of persistent high or low health literacy (e.g., using the growth-based trajectory modelling method) on health outcomes over the life course.
The pathway model	To examine the pathway effects whereby early experiences set in motion a chain of events that put individuals on paths differentiated by types and levels of exposures to social and biological factors [65].	To examine the mediating role of health literacy (e.g., adolescent health literacy) in the relationship between socioeconomic disadvantage and health outcomes.
The social mobility model	To examine the unique importance of social mobility in explaining the early-life and later-life socioeconomic status and health link [66].	To examine whether the effect of later-life exposure (e.g., socioeconomic status, immigration status) on health literacy is stronger than the effect of early-life exposure.

**Table 2:** Applying open science to health literacy research and practice.

Action Area	Health Literacy at Different Levels(Individual, Organization, National, and Global)
**Preregistration**	A preregistered protocol for interventional studies, such as randomized controlled trials or quasi-experimental research (e.g., ClinicalTrials.gov, International Clinical Trials Registry Platform) Use the LifeCourse analysis plan template (https://doi.org/10.26188/12471380) to build a research proposal and refine data analytic approach for observational studies A focus on the prespecified hypothesis rather than hypothesis driven from post hoc data analyses
**Open materials**	Make documentation of materials and data explicit and easy-to-find in the directory Make health literacy measurement tools available on the public repository with corresponding documentation (e.g., Health Literacy Tool Shed) Make the components of the research methodology publicly available for others to reproduce the reported procedure and analyses Document all study variables in a separate spreadsheet, including label description and response options. Write annotated do-files for dataset creation, variable creation and data analysis, including decisions about cut-offs and relevant references Share data coding on an open-access repository with corresponding documentation (e.g., Open Science Framework, Figshare) Document major deviations from the analysis plan in a data analysis log Make the publications open access for the public, including additional materials (e.g., variable description, additional analyses) in the supplementary files when required
**Open data**	Be clear about data security policy about participants’ privacy and confidentiality Have a fully traceable path from the source dataset to the paper working dataset Include “data availability statement” in publications even if data sharing is not possible or advisable Make health literacy data available on an open-access repository with corresponding documentation (e.g., Open Science Framework, Figshare) whenever possible Report statistical results according to “ATOMIC” recommendations: accept uncertainty, be thoughtful, open, and modest, and institutional change
